# Episodic memory trajectories of older adults with and without HIV: A longitudinal population-based study in rural South Africa

**DOI:** 10.1371/journal.pgph.0006572

**Published:** 2026-06-26

**Authors:** Michaela Theilmann, Sarah Gao, Darina T. Bassil, Ryan G. Wagner, Xuexin Yu, Eric Tchetgen Tchetgen, Molly Rosenberg, Carla Roberts-Toler, Brendan Maughan-Brown, Lindsay Kobayashi, Charlotte Kelham, Kathleen Kahn, John Joska, Guy Harling, Jose Gutierrez, Chido Chinogurei, Julia K. Rohr, Jennifer Manne-Goehler

**Affiliations:** 1 Division of Infectious Diseases, Brigham and Women’s Hospital, Harvard Medical School, Boston, Massachusetts, United States of America; 2 Medical Practice Evaluation Center, Massachusetts General Hospital, Harvard Medical School, Boston, Massachusetts, United States of America; 3 Center for Economic and Social Research, University of Southern California, Los Angeles, California, United States of America; 4 Harvard Center for Population and Development Studies, Harvard University, Cambridge, Massachusetts, United States of America; 5 SAMRC/Wits Rural Public Health and Health Transitions Research Unit (Agincourt), School of Public Health, Faculty of Health Sciences, University of the Witwatersrand, Johannesburg, South Africa; 6 Department of Neurology, Barrow Neurological Institute, Phoenix, Arizona, United States of America; 7 Department of Epidemiology, Columbia University Mailman School of Public Health, New York, New York, United States of America; 8 Department of Statistics and Data Science, The Wharton School, University of Pennsylvania, Philadelphia, Pennsylvania, United States of America; 9 Department of Epidemiology and Biostatistics, Indiana University School of Public Health-Bloomington, Bloomington, Indiana, United States of America; 10 Southern Africa Labour and Development Research Unit, University of Cape Town, Cape Town, South Africa; 11 Department of Epidemiology, University of Michigan School of Public Health, Ann Arbor, Michigan, United States of America; 12 Institute for Global Health, University College London, London, United Kingdom; 13 University of Cape Town, HIV Mental Health Research Unit, Division of Neuropsychiatry, Department of Psychiatry and Mental Health, Cape Town, South Africa; 14 Africa Health Research Institute, KwaZulu-Natal, South Africa; 15 School of Nursing & Public Health, College of Health Sciences, University of KwaZulu-Natal, Durban, South Africa; 16 Department of Neurology, Miller School of Medicine, University of Miami, Miami, Florida, United States of America; 17 Centre for Integrated Data and Epidemiological Research, School of Public Health, University of Cape Town, Cape Town, South Africa; 18 Heidelberg Institute of Global Health, Heidelberg University, Heidelberg Germany; PLOS: Public Library of Science, UNITED STATES OF AMERICA

## Abstract

With the introduction of antiretroviral treatment (ART), life expectancy among people with HIV in South Africa has increased substantially. This ageing population faces age-related challenges, including cognitive decline. Yet, differences in cognitive trends between people with and without HIV remain poorly understood. We analyzed longitudinal data on 3,066 adults aged 40 + years in rural South Africa from the “Health and Aging in Africa: Longitudinal Studies in South Africa" (HAALSA) cohort. Using generalized estimating equations, we compared episodic memory trajectories over seven years (2014 – 2022) across three groups: (1) people without HIV, (2) people with HIV and suppressed viral load, and (3) people with HIV and unsuppressed viral load. Furthermore, we predicted episodic memory scores at yearly intervals. On average, the trend in episodic memory decline did not differ between people without HIV and people with HIV with suppressed or unsuppressed viral load. However, looking individually at yearly intervals revealed that participants without HIV experienced significantly steeper episodic memory decline compared to participants with HIV with suppressed viral load when controlling for individual-level characteristics. Consequently, over the seven-year period, participants without HIV progressed from having episodic memory comparable to those with suppressed viral load to levels similar to those with unsuppressed viral load. The slower episodic memory decline among virally suppressed people with HIV compared to those without HIV may reflect benefits from regular healthcare engagement or may also be a result of selective mortality. Our findings suggest that policymakers should: (1) intensify efforts to increase ART coverage and adherence among unsuppressed people with HIV; (2) implement targeted cognitive screening for at-risk individuals regardless of HIV status; (3) expand screening and treatment for conditions that contribute to cognitive decline; and (4) promote behaviors that may preserve cognitive function, particularly in resource-constrained settings. Further research on potential underlying mechanisms should be conducted.

## Introduction

The global landscape of HIV has been transformed by modern antiretroviral therapy (ART), shifting HIV from a fatal diagnosis to a manageable chronic condition with substantially increased life expectancy [1–4]. This remarkable achievement has brought new challenges: as people with HIV age, they face an increasing burden of age-related health conditions, including cognitive decline [5]. The intersection of HIV, aging, and cognition presents a critical public health challenge, particularly in high-prevalence settings. Cognitive disorders in people with HIV continue to be prevalent, even in the era of effective ART [6,7]. In most cases, the damage is caused prior to ART initiation, where “legacy effects” prevail [7]. In a very small group, central nervous system (CNS) escape may occur and the effects of low-grade chronic inflammatory CNS changes are unknown [8,9]. While ART has dramatically reduced the incidence of severe forms like HIV-associated dementia, the presence of legacy damage, together with persisting low-grade inflammation, and the potential neurotoxic effects of some antiretroviral drugs, raise important questions about long-term cognitive health in people with HIV [10,11].

These concerns are particularly pressing in South Africa, where 17% of the population aged 15 years and older lives with HIV [12]. Despite impressive progress in ART coverage (74%) and viral suppression (67%), the country still faces a complex public health challenge: as life expectancy has increased from 53.6 to 66.5 years over the past two decades with many more living with HIV into older age, the intersection of HIV and neurocognitive health is becoming increasingly an important consideration. Moreover, the South African health system is not prepared for this demographic shift [13]. Understanding this intersection is crucial for healthcare planning and policy-making, yet evidence on cognitive function and trajectories among people with HIV with and without viral suppression remains limited [14–18]. Specifically, it is not well understood yet whether legacy effects and low-grade inflammation contribute to accelerated aging in people with HIV compared to those without [19–21]. As the population of people with HIV is rapidly aging, policymakers and healthcare providers need a robust evidence base to assess whether and to what extent targeted cognitive intervention and care is required.

Our study addresses this critical knowledge gap by examining trajectories of episodic memory, one of the cognitive function domains, in a longitudinal cohort (2014–2022) of adults aged 40 years and older in rural South Africa. Using data from the “Health and Aging in Africa: Longitudinal Studies in South Africa" (HAALSA) project, we compare changes in episodic memory over time across three groups: (1) people with HIV with suppressed viral load, (2) people with HIV with unsuppressed viral load, and (3) people without HIV. Our analysis provides essential insights for developing targeted interventions and healthcare strategies in aging populations with HIV, both in South Africa and other high-prevalence settings.

## Methods

### Ethical approval

Ethical approval for HAALSA was obtained from the University of the Witwatersrand Human Research Ethics Committee (no. M141159), the Harvard T.H. Chan School of Public Health Office of Human Research Administration (no. 13-1608), and the Mpumalanga Provincial Research and Ethics Committee.

### Data

We used data from the HAALSA cohort, formerly known as “The Health and Aging in Africa: A Longitudinal Study of an INDEPTH Community in South Africa” (HAALSI), which is a comprehensive research project studying aging, health, and well-being in a rural South African community [22]. It is set in the Agincourt Health and Socio-Demographic Surveillance System (HDSS) site in rural Mpumalanga province, north-eastern South Africa. The study area is characterized by limited infrastructure development and significant socioeconomic challenges, with many households relying on government pensions and experiencing food shortages [23]. Educational attainment is low, given the legislated limits on educational opportunities placed on the Black South Africans during the Apartheid era, 1948–1993. Almost half of participants reported no formal schooling, and unemployment affected approximately three-quarters of the study population [22]. The HAALSA sample is a gender-stratified random sample of adults aged 40 years and older living in the Agincourt HDSS [22]. The HDSS covered 31 villages with a combined population of 116,000. Of the 12,875 eligible individuals (aged 40 years and older and having lived in the HDSS for at least 12 months), 6,281 participants were randomly selected, of which 5,059 (2,714 women; 2,345 men) completed the interview [22]. The survey instrument combines biological measurements, including HIV testing and anthropometrics, with detailed questionnaires covering socioeconomic conditions, health behaviors, medical history, and cognitive assessments. The survey instruments were translated to and interviews were conducted in Shangaan, the local language spoken in Agincourt. Written informed consent was obtained from all potential participants. If a participant could not read, they signed the consent form with a fingerprint, while a witness was present [22]. We used longitudinal data from three HAALSA waves (November 13, 2014 – November 30, 2015, October 12, 2018 – November 7, 2019, and July 27, 2021 – March 3, 2022).

### Outcome

Each HAALSA survey administered a brief, in-person cognitive assessment harmonized with that used in the US Health and Retirement Study, which assessed orientation, episodic memory, and numeracy. We used the measure of episodic memory as the outcome for this analysis, due to the sensitivity of episodic memory to both HIV- and aging-related changes and because it is a hallmark early symptom of dementia [24]. Episodic memory was assessed as the immediate and delayed verbal recall of a list of ten nouns read out loud by the study interviewer. We conducted a confirmatory factor analysis to harmonize longitudinal episodic memory factor scores at each time point, due to changes in the administration of the episodic memory tests across waves (one immediate recall test in Wave 1; three immediate recall tests in Waves 2–3). The episodic memory scores were standardized to the Wave 1 factor distribution, with higher scores indicating higher memory function. Thus, the regression coefficients express point estimates in the unit of the standard deviation of the baseline distribution. Further details on the episodic memory measures and the calculation of the score can be found elsewhere [25,26].

### Exposure

Our primary exposure variable was HIV status, determined at Wave 1 through ELISA testing of dried blood spots (Vironostika Uniform 11, Biometrica, France). For HIV-positive participants, viral load was measured using Biomeriux NucliSens (Durham, NC, USA), with viral suppression defined as <200 copies/mL [22]. Based on these measurements, we categorized participants into three groups: HIV-negative, HIV-positive with viral suppression, and HIV-positive without viral suppression. If HIV status was not determined at Wave 1, we took a last value carry forward approach and used the HIV status from Wave 2 (n = 64) or Wave 3 (n = 49).

### Confounding variables

We included a range of potential confounding variables measured at the baseline in our regression models:

*Socio-demographic characteristics:* Age, sex (male; female), education [no formal education; some primary (1–7 years); some secondary (8–11 years); secondary or more (12 + years)], literacy (can read and write; cannot read and write), country of origin (South Africa; Mozambique or other countries), currently married (yes; no), currently working (yes; no), and household wealth quintile based on an asset index calculated with a Principal Component Analysis [27]. The asset index is based on self-reported information on the ownership of assets and livestock and housing characteristics.

*Behavioral risk factors*: current smoker (yes; no) and alcohol consumption during the past 30 days (yes; no).

*Health*: Presence of depressive symptoms was assessed with the 8-item Center for Epidemiologic Studies-Depression (CES-D) scale [28]. The score was included as a continuous variable ranging from 0 to 8 with higher values indicating presence of more depressive symptoms. Individuals were defined to have hypertension if they reported currently taking hypertension medication or had a blood pressure reading above 140/90mmHg. Similarly, diabetes status was defined based on self-report of current diabetes medication use or a blood glucose level of ≥ 7 mmol/l for fasting individuals and ≥ 11.1 mmol/l for non-fasting individuals. We also included visit to a public or private health facility in the past three months (yes; no) and self-rated health during childhood (very good/good; moderate/bad/very bad).

### Statistical analysis

Our analytical approach comprised three main steps. First, we presented baseline sample characteristics. Second, we estimated generalized estimating equations (GEE) with a Gaussian distribution family and identity link function, independent correlation structure, and robust standard errors clustered at the individual-level [29,30]. We estimated a GEE rather than a mixed effects model as the independent correlation structure yields unbiased estimates even if attrition is selective. We specified two regression models: Model 1: Adjusted for age; Model 2: Adjusted for all potential confounders listed above. All models accounted for practice effects, which typically occur when cognitive assessments are repeated, with the largest improvement usually observed between the first and second assessments [31,32]. We controlled for this by including a binary indicator for first episodic memory testing occasion as a fixed effect [33]. To assess episodic memory trajectories over time, we included time in years since baseline as a continuous variable and interacted it with the HIV and viral suppression status exposure (HIV-negative, HIV-positive with viral suppression, and HIV-positive without viral suppression), allowing us to examine whether slopes of episodic memory differed across the three groups. Finally, we estimated predicted episodic memory scores at each year of follow-up by HIV and viral suppression status. To address potential bias from mortality and attrition between Waves 1 and 3, we applied mortality and attrition-adjusted sampling weights throughout our analyses [30]. The inverse probability weights for mortality and attrition were constructed separately and then the product was used as final weight [34]. We only included complete cases, and results are presented with 95% confidence intervals and corresponding p-values. All analyses were conducted in Stata 17.

### Sensitivity analyses

We conducted several sensitivity analyses to test the robustness of our results. First, rather than defining HIV status at baseline, we considered that some individuals transitioned between statuses. Hence, we defined an alternative exposure that captures HIV status across all waves: always negative, always suppressed, at times or always unsuppressed, and converter from HIV negative to positive. Second, we estimated a mixed effects model with random intercepts. Unlike GEE, mixed models cannot incorporate inverse probability weights, and therefore rely on the assumption that data are Missing at Random conditional on observed covariates. Third, we additionally included a continuous post-traumatic stress disorder score and an emotional well-being score as control variables [35,36]. Details on how these two scores were generated are provided in S1 Table.

## Results

**Fig 1:**
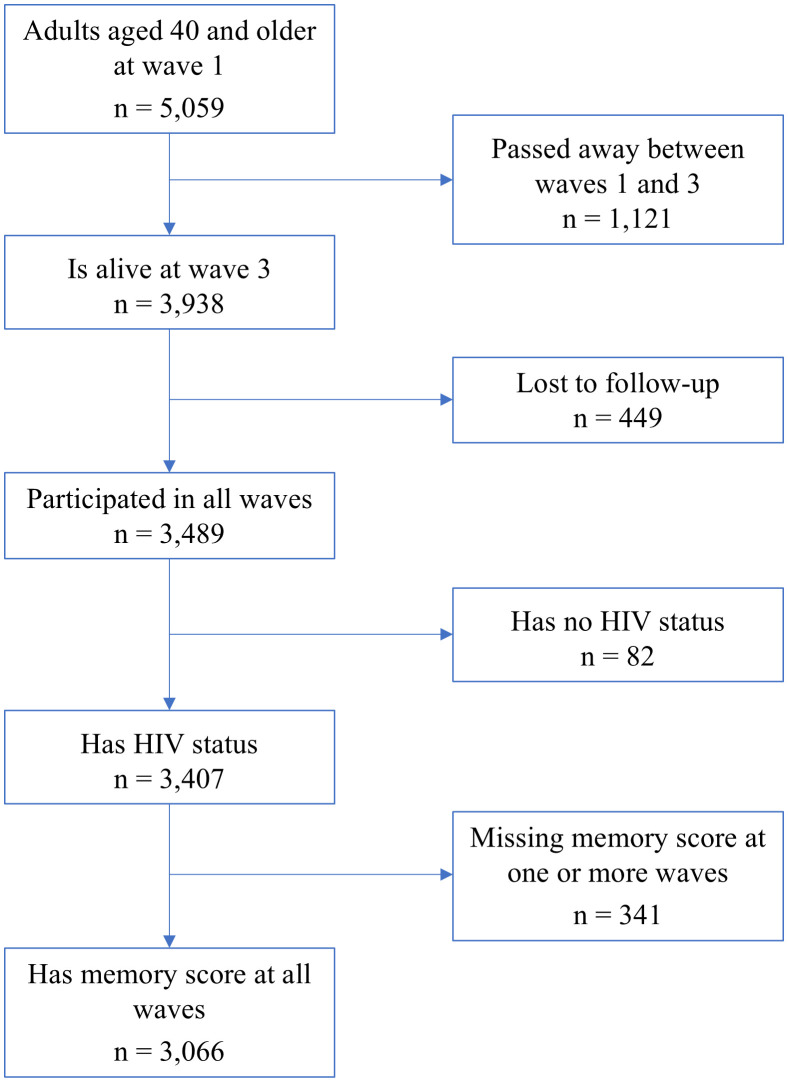
Sample inclusion and exclusion.

5,509 adults aged 40 years or older participated in the Wave 1 interview (Fig 1). 1,121 passed away between Waves 1 and 3, and 449 were lost to follow-up for other reasons. Furthermore, we excluded 82 individuals who did not have data on HIV status and 341 who did not have episodic memory scores at all three waves, resulting in an analytical dataset with data on 3,066 individuals.

### Sample characteristics

At Wave 1, 58.4% of respondents included in our sample were female and the mean age was 59.9 years (SD: 11.6) (Table 1). 55.3% of participants reported being literate and just under half of individuals had no formal education (41.5%). Approximately half of the sample was married (54.5%). Survey participants had a score of 1.3 on the CES-D 8 scale on average, 62.2% had hypertension, and 10.0% had diabetes. 88.0% reported very good or good childhood health and 44.6% reported having used a healthcare facility in the past three months.

**Table 1:** Sample characteristics.

	All	Negative	Positive, suppressed	Positive, unsuppressed	Group difference
**Socio-demographic characteristics**					
Female [N (%)]	1,791 (58.4%)	1,348 (58.4%)	278 (59.0%)	165 (57.5%)	0.917
Age [mean (SD)]	59.9 (11.6)	61.4 (11.7)	55.7 (9.6)	54.1 (9.7)	<0.001
Literate [N (%)]	1,695 (55.3%)	1,253 (54.3%)	284 (60.3%)	158 (55.2%)	0.059
*Education [N (%)]*					
No education	1,268 (41.5%)	978 (42.5%)	175 (37.2%)	115 (40.5%)	0.007
Primary (1–7 years)	1,126 (36.8%)	850 (36.9%)	181 (38.4%)	95 (33.5%)	
Secondary (8–12 years)	385 (12.6%)	261 (11.3%)	72 (15.3%)	52 (18.3%)	
Secondary or more (12 + years)	279 (9.1%)	214 (9.3%)	43 (9.1%)	22 (7.7%)	
Born in South Africa [N (%)]	2,130 (69.5%)	1,632 (70.7%)	327 (69.6%)	171 (59.8%)	<0.001
*Wealth quintile [N (%)]*					
Quintile 1	591 (19.3%)	421 (18.2%)	92 (19.5%)	78 (27.2%)	<0.001
Quintile 2	587 (19.1%)	432 (18.7%)	94 (20.0%)	61 (21.3%)	
Quintile 3	598 (19.5%)	445 (19.3%)	98 (20.8%)	55 (19.2%)	
Quintile 4	647 (21.1%)	485 (21.0%)	106 (22.5%)	56 (19.5%)	
Quintile 5	643 (21.0%)	525 (22.7%)	81 (17.2%)	37 (12.9%)	
Married [N (%)]	1,670 (54.5%)	1,362 (59.0%)	185 (39.3%)	123 (43.0%)	<0.001
**Behavioral risk factors**					
Current smoker [N (%)]	256 (8.4%)	183 (7.9%)	46 (9.8%)	27 (9.4%)	0.338
Consumes alcohol [N (%)]	664 (21.7%)	506 (21.9%)	94 (20.0%)	64 (22.3%)	0.616
**Health**					
CES-D 8 score [mean (SD)]	1.3 (1.5)	1.4 (1.5)	1.2 (1.4)	1.2 (1.5)	0.038
Hypertension [N (%)]	1,896 (62.2%)	1,538 (67.0%)	226 (48.5%)	132 (46.0%)	<0.001
Diabetes [N (%)]	290 (10.0%)	238 (10.9%)	33 (7.4%)	19 (6.9%)	0.017
Good childhood health [N (%)]	2,696 (88.0%)	2,034 (88.1%)	408 (86.6%)	254 (88.8%)	0.591
Health service use [N (%)]	1,342 (44.6%)	915 (40.5%)	304 (65.2%)	123 (43.6%)	<0.001
**Z-standardized episodic memory score**					
Wave 1 (2014–15) [mean (SD)]	0.1 (1.0)	0.1 (1.0)	0.2 (0.9)	0.1 (1.0)	0.027
*N*	3,066 (100.0)	2,308 (75.3)	471 (15.4)	287 (9.4)	

Note: Group differences in continuous variables were determined through linear regression. Group differences in binary and categorical variables were determined through the Pearson’s chi-squared test.

Abbreviations: CES-D: Center for Epidemiologic Studies-Depression; SD = standard deviation

The HIV prevalence in our study sample was 26.2%. Participants with HIV were, on average, six years younger than individuals without HIV and tended to have a lower socio-economic status. They also had a lower hypertension prevalence compared to their HIV negative peers.

### Regression results

In Model 1, which controls for age and practice effects, the main effects of the HIV status showed that the episodic memory score at baseline among unsuppressed people with HIV (β = -0.173 SD units; 95% CI -0.290, -0.056) was lower than among individuals without HIV, and did not statistically differ from the episodic memory score among people with HIV and suppressed viral load (β = 0.001 SD units; 95% CI -0.094, 0.096) (Table 2).

**Table 2:** Coefficients from generalized estimation equation regression models.

	Model 1	Model 2
	β [95% CI] *p-value*	β [95% CI] *p-value*
**HIV Status**		
Negative	*Ref.*	*Ref.*
Positive, suppressed	0.001	0.037
	[-0.094, 0.096]	[-0.046, 0.119]
	*0.98*	*0.39*
Positive, unsuppressed	-0.173	-0.092
	[-0.290, -0.056]	[-0.208, 0.023]
	*< 0.001*	*0.13*
**Years since Wave 1**		
	-0.034	-0.033
	[-0.048, -0.020]	[-0.048, -0.019]
	*< 0.001*	*< 0.001*
**HIV status x Years since Wave 1**		
Negative x Months	*Ref.*	*Ref.*
Positive, suppressed x Months	0.005	0.013
	[-0.014, 0.023]	[-0.002, 0.028]
	*0.63*	*0.08*
Positive, unsuppressed x Months	0.013	0.015
	[-0.009, 0.035]	[-0.009, 0.038]
	*0.24*	*0.22*
**Control variables**		
Age	x	x
Practice effects	x	x
Socio-demographic		x
Behavioral		x
Comorbidities		x
*N*	*9,198*	*8,511*

Note: The table displays the main effect estimates of HIV status and interaction estimates with years since baseline. 95% confidence intervals are displayed in squared brackets and p-values in italics. S1 Table is the full regression table displaying coefficients, confidence intervals, and p-values for all variables included in the regression model.

Abbreviations: CI = confidence interval; Ref = reference category.

The coefficient for people with HIV and suppressed viral load remained small and not significant when controlling for potential confounders (Model 2). The main point estimate for people with HIV and unsuppressed viral load decreased in magnitude and turned insignificant when including control variables (Tables 2 and S2).

The coefficients of the interaction effects represent differences in the overall rate of change in episodic memory scores across HIV status. Relative to the HIV negative group, the rate of change in memory scores was not significantly different for either of the HIV positive groups with and without viral suppression [β = 0.005 SD units/year; 95% CI -0.014, 0.023, and β = 0.013 SD units/year; 95% CI -0.009, 0.035, respectively] (Table 2). Including additional control variables in the regression model yielded similar results.

**Fig 2:**
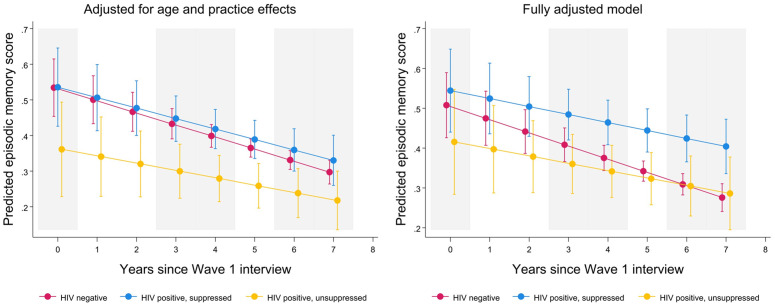
Predicted episodic memory scores. This figure displays the predicted episodic memory score resulting from generalized estimating equations with 95% confidence intervals at yearly intervals for the three HIV status groups. Survey data were collected in the grey-shaded years.

To assess whether the relative differences in memory function by HIV and viral suppression status changed over time, we estimated the predicted values of the episodic memory score over time by HIV/viral suppression status (Fig 2, S3 Table). We found that episodic memory scores were comparable among individuals without HIV and those with HIV and suppressed viral load when controlling only for age and the practice effect. However, when controlling for additional individual-level characteristics, the trends diverged. From year 5 onwards, people with HIV with a suppressed viral load had statistically significant higher episodic memory scores than those without HIV. There was substantial uncertainty in the estimation of predicted episodic memory scores of the HIV positive and unsuppressed group, leading to wide confidence intervals. However, it seems that predicted episodic memory scores were lower among people with HIV with unsuppressed viral load compared to those with suppressed viral load.

### Sensitivity analyses

Our results were robust to changes in model specifications. When considering changes in HIV status across waves, we found slightly different coefficient estimates, but the overall conclusions of the main analysis remained valid (S1 Fig). Estimating a mixed effects model with random intercepts also did not alter the results (S2 Fig) nor did including two additional mental health indicators (S4 Table, S3 Fig).

## Discussion

Our study revealed an unexpected and complex relationship between HIV status and episodic memory decline over time amongst middle-aged and older adults living in rural South Africa. While regression analyses showed no statistically significant differences in episodic memory trajectories when averaged over the years, our detailed temporal analyses uncovered a striking pattern: HIV-negative participants’ steeper episodic memory decline meant that over a seven-year period they moved from having episodic memory scores comparable to counterparts living with suppressed HIV viral loads to that of those living with unsuppressed HIV.

Previous studies mainly focus on cognitive impairment among people with HIV and whether ART halts or reverses the progress of HIV-associated neurocognitive disorder, as well as comparing people without HIV to people with HIV, ignoring potential differences between individuals with and without successful HIV management through ART [37–39]. Studies comparing people with HIV and suppressed viral load, people with HIV and unsuppressed viral load, and individuals without HIV over time are scarce [16, 40–44]. Our results are in line with a longitudinal study using data from the US Women’s Interagency HIV Study (WIHS), which shows that the decline in memory among women without HIV over four years is steeper than among women with HIV and suppressed viral load [43].

There are several potential mechanisms that might explain the slower decline in episodic memory among people with HIV with suppressed viral load compared to individuals without HIV. First, people with HIV are tightly linked to the health care system for regular check-ups and disease monitoring. This regular engagement with healthcare services means that cognitive decline and other conditions affecting cognition, such as cardiometabolic diseases, might be detected and treated earlier [45]. Furthermore, due to their continuous linkage to care, people with HIV might be more health literate and, over the life course, develop behavioral patterns that protect against cognitive decline. Second, while ART has been shown to improve cognition, this relationship appears complex [37]. The regular monitoring and management of inflammation and immune function in people with HIV might provide additional cognitive benefits beyond viral suppression, though evidence on whether these effects may offset the negative neurocognitive impact of HIV infection itself remains mixed [20]. Third, our results may be influenced by survival patterns resulting from South Africa’s delayed ART rollout. The government’s implementation of ART began only in 2004, leading to an estimated 330,000 preventable deaths between 2000 and 2005 [46]. In the Agincourt area, which we studied, ART was available at the large scale even later [47,48]. This timing is particularly relevant for our study population, who were 40 years or older at the HAALSA baseline in 2014. Study participants with HIV, thus, represent the subset of individuals with HIV who survived the pre-ART era. These survivors may have underlying health advantages that distinguish them from their HIV-negative peers, potentially influencing our observed differences in episodic memory decline.

Our results yield important insights for policymakers. First, given the substantial and persistent cognitive gap between people with HIV who had suppressed or non-suppressed viral loads, policymakers should intensify their efforts to increase ART coverage and adherence among people with HIV who are not on ART or have not achieved viral suppression. This could ultimately benefit a large proportion of the population with untreated HIV at risk for poor cognitive outcomes. Second, health services and screening for cognitive impairment and its risk factors should be made more accessible to everyone, including people without HIV. Cognitive tests should be integrated into routine care for middle-aged and older adults and conducted at regular intervals to ensure early detection and treatment. Making cognitive tests universally available might not be feasible in resource constrained settings. Targeting at-risk individuals, for example those with self-reported symptoms of cognitive decline or with a higher vascular risk, can lead to early detection even if resources are limited. Third, screening and treatment of diseases that may lead to cognitive decline, such as cardiometabolic diseases, should be expanded and cover the entire population irrespective of their HIV status. Fourth, healthy behaviors that may prevent cognitive decline, such as a healthy diet and physical activity, should be promoted – in particular in resource constrained settings, where treatments may not be readily available.

Our study has several strengths. First, we had a long follow-up period of almost 10 years, which allowed us to detect emerging differences in episodic memory trajectories that might have been missed in shorter studies. This long-term follow-up allowed us to see the significant implications of the accumulation of small differences in episodic memory across the years. Second, we had data on individuals from age 40 upwards – the age range that is particularly susceptible to cognitive decline as they age. Third, we had information on individuals’ HIV and viral suppression status, allowing us to compare not only individuals with and without HIV but also to distinguish between individuals with suppressed and unsuppressed viral load.

Our study also has some limitations. First, we were only able to assess changes in episodic memory, whereas cognition has many other domains, such as executive function and verbal fluency, some of which are more specific to HIV-associated neurocognitive diseases and time trends might differ between these domains. Thus, our results should not be generalized to cognitive abilities in general [37,43]. Second, there is a survivorship bias such that the fittest people with HIV survived to enter the HAALSA cohort. We do apply sampling weights that account for differential mortality post-baseline, but this adjustment likely does not account for pre-baseline selection. Third, our results may be specific to Agincourt, a rural community with low education and high rates of unemployment and poverty. Thus, our findings may not be generalizable to other contexts within and outside South Africa [49]. Fourth, we do not have information on the time point of HIV diagnosis, ART adherence, and viral suppression over the years, which may influence cognitive abilities.

More research should be done comparing trends in cognition separately for individuals without HIV, with suppressed viral load, and with unsuppressed viral load. These studies should also consider a broader set of cognitive domains and assess potential differences across these. Furthermore, studies should investigate how health services for cognition and prevention of cognitive decline can be integrated into existing health service structures, including in resource-constrained settings. Another important aspect is to study how health service coverage for HIV and other diseases affecting cognition can be extended to cover all middle-aged and older adults, which becomes particularly relevant and challenging in aging societies.

## Conclusion

Our findings highlight the complex interplay between HIV status and episodic memory trajectories over time. The steeper episodic memory decline among participants without HIV compared to those with HIV who have suppressed viral loads presents important implications for health policy. Expanding ART coverage and adherence support, broadening the focus to the general ageing population, and implementing prevention measures are all essential for reducing episodic memory decline at the population level. As societies age globally, ensuring access to such services becomes increasingly important for maintaining cognitive health in later life.

## Supporting information

S1 Table Indicator construction of post-traumatic stress disorder and emotional well-being. (DOCX)

S2 Table Full regression table. (DOCX)

S3 Table Marginal effects. (DOCX)

S4 Table Regression coefficients of Model 2 with two additional mental health covariates. (DOCX)

S1 Fig HIV status definition across survey waves. (DOCX)

S2 Fig Mixed-effects model. (DOCX)

S3 Fig Model 2 with two additional mental health covariates. (DOCX)
