# Supplementary material for: Episodic memory trajectories of older adults with and without HIV: A longitudinal population-based study in rural South Africa
Source: PLOS Glob Public Health. 2026 Jun 26;6(6):e0006572. doi: 10.1371/journal.pgph.0006572 (PMC13309049; doi:10.1371/journal.pgph.0006572)
Supplement: S1 Table — (DOCX) [file pgph.0006572.s001.docx]

S1 Table: Indicator construction of post-traumatic stress disorder and emotional well-being

| Post-traumatic stress disorder score | In your life, have you ever had any experience that was so frightening, horrible, or upsetting that, in the past 30 days you…  a) avoided being reminded of this experience by staying away from certain places, people or activities?  b) lost interest in activities that were once important or enjoyable?  c) began to feel more isolated or distant from other people?  d) found it hard to have love or affection for other people?  e) began to feel that there was no point in planning for the future?  f) had more trouble than usual falling asleep or staying asleep?  g) became jumpy or got easily startled by ordinary noises or movements? | Each “yes” answer adds 1 point to the score. |
| --- | --- | --- |
| Emotional well-being | a) Did you feel well-rested yesterday?  b) Were you treated with respect all day yesterday?  c) Did you smile or laugh a lot yesterday?  d) Did you learn or do something interesting yesterday?  e) Did you experience the following feelings during a lot of the day yesterday? How about enjoyment?  f) Did you experience the following feelings during a lot of the day yesterday? How about physical pain? g) Did you experience the following feelings during a lot of the day yesterday? How about worry?  h) Did you experience the following feelings during a lot of the day yesterday? How about sadness?  i) Did you experience the following feelings during a lot of the day yesterday? How about stress?  j) Did you experience the following feelings during a lot of the day yesterday? How about anger? | Each “yes” answer to questions a) to e) adds 1 point to the score. Each “no” answer to questions f) to j) adds 1 point to the score. |
