# Supplementary material for: Episodic memory trajectories of older adults with and without HIV: A longitudinal population-based study in rural South Africa
Source: PLOS Glob Public Health. 2026 Jun 26;6(6):e0006572. doi: 10.1371/journal.pgph.0006572 (PMC13309049; doi:10.1371/journal.pgph.0006572)
Supplement: S2 Table — (DOCX) [file pgph.0006572.s002.docx]

S2 Table: Full regression table

|  | **Model 1** | **Model 2** |
| --- | --- | --- |
| **HIV Status** | | |
| Negative | *Ref.* | *Ref.* |
| Positive, suppressed | 0.001 | 0.037 |
|  | [-0.094, 0.096] | [-0.046, 0.119] |
|  | *0.98* | *0.39* |
| Positive, unsuppressed | -0.173 | -0.092 |
|  | [-0.290, -0.056] | [-0.208, 0.023] |
|  | *<0.001* | *0.12* |
| **Years since Wave 1** | | |
|  | -0.034 | -0.033 |
|  | [-0.048, -0.020] | [-0.048, -0.019] |
|  | *<0.001* | *<0.001* |
| **HIV status x Years since Wave 1** | | |
| Negative x Months | *Ref.* | *Ref.* |
| Positive, suppressed x Months | 0.005 | 0.013 |
|  | [-0.014, 0.023] | [-0.002, 0.028] |
|  | *0.63* | *0.08* |
| Positive, unsuppressed x Months | 0.013 | 0.015 |
|  | [-0.009, 0.035] | [-0.009, 0.038] |
|  | *0.24* | *0.22* |
| **Continuous control variables** | | |
| Age | -0.028 | -0.019 |
|  | [-0.030, -0.026] | [-0.022, -0.017] |
|  | *<0.001* | *<0.001* |
| CESD-8 Score |  | -0.019 |
|  |  | [-0.033, -0.006] |
|  |  | *0.01* |
| **Practice effect** | | |
| No | *Ref.* | *Ref.* |
| Yes | -0.472 | -0.467 |
|  | [-0.551, -0.392] | [-0.549, -0.384] |
|  | *<0.001* | *<0.001* |
| **Sex** | | |
| Male |  | *Ref.* |
| Female |  | -0.027 |
|  |  | [-0.075, 0.021] |
|  |  | *0.27* |
| **Education** | | |
| No education |  | *Ref.* |
| Primary (1-7 yr) |  | 0.080 |
|  |  | [0.022, 0.137] |
|  |  | *0.01* |
| Secondary (8-12 yr) |  | 0.249 |
|  |  | [0.166, 0.332] |
|  |  | *<0.001* |
| Secondary or more (12+ yr) |  | 0.408 |
|  |  | [0.311, 0.504] |
|  |  | *<0.001* |

| **Wealth quintile** | | |
| --- | --- | --- |
| Quintile 1 |  | *Ref.* |
| Quintile 2 |  | -0.015 |
|  |  | [-0.081, 0.050] |
|  |  | *0.64* |
| Quintile 3 |  | 0.054 |
|  |  | [-0.015, 0.122] |
|  |  | *0.12* |
| Quintile 4 |  | 0.076 |
|  |  | [0.010, 0.141] |
|  |  | *0.02* |
| Quintile 5 |  | 0.121 |
|  |  | [0.050, 0.193] |
|  |  | *<0.001* |
| **Marital status** | | |
| Not married |  | *Ref.* |
| Married |  | 0.058 |
|  |  | [0.014, 0.102] |
|  |  | *0.01* |
| **Current smoker** | | |
| No |  | *Ref.* |
| Yes |  | -0.035 |
|  |  | [-0.116, 0.046] |
|  |  | *0.39* |
| **Consumes alcohol** | | |
| No |  | *Ref.* |
| Yes |  | -0.100 |
|  |  | [-0.157, -0.044] |
|  |  | *<0.001* |
| **Has hypertension** | | |
| No |  | *Ref.* |
| Yes |  | 0.014 |
|  |  | [-0.029, 0.057] |
|  |  | *0.53* |
| **Has diabetes** | | |
| No |  | *Ref.* |
| Yes |  | -0.047 |
|  |  | [-0.115, 0.022] |
|  |  | *0.18* |
| **Literate** | | |
| No |  | *Ref.* |
| Yes |  | 0.216 |
|  |  | [0.162, 0.270] |
|  |  | *<0.001* |
| **Born in South Africa** | | |
| No |  | *Ref.* |
| Yes |  | 0.058 |
|  |  | [0.006, 0.109] |
|  |  | *0.03* |
| **Self-rated child health** | | |
| Very good, good |  | *Ref.* |
| Moderate, bad, very bad |  | 0.153 |
|  |  | [0.094, 0.211] |
|  |  | *<0.001* |
| **Health service use** | | |
| No |  | *Ref.* |
| Yes |  | -0.104 |
|  |  | [-0.145, -0.062] |
|  |  | *<0.001* |
| **Intercept** | | |
|  | 2.272 | 1.360 |
|  | [2.130, 2.414] | [1.176, 1.543] |
|  | *<0.001* | *<0.001* |
| ***N*** | ***9,198*** | ***8,511*** |

*Note: The table displays the coefficients of the generalized estimation equation regression models. 95% confidence intervals are displayed in squared brackets and p-values in italics.*

*Abbreviations: CES-D: Center for Epidemiologic Studies-Depression; CI = confidence interval; Ref = reference category*
