# Supplementary material for: Episodic memory trajectories of older adults with and without HIV: A longitudinal population-based study in rural South Africa
Source: PLOS Glob Public Health. 2026 Jun 26;6(6):e0006572. doi: 10.1371/journal.pgph.0006572 (PMC13309049; doi:10.1371/journal.pgph.0006572)
Supplement: S3 Table — (DOCX) [file pgph.0006572.s003.docx]

S3 Table: Marginal effects

|  | Model 1 | Model 2 |
| --- | --- | --- |
| **Year # HIV status** | Marginal effect  [95% CI] | Marginal effect  [95% CI] |
| # Negative | 0.534 | 0.508 |
|  | [0.454, 0.615] | [0.426, 0.589] |
| # Positive, suppressed | 0.536 | 0.544 |
|  | [0.426, 0.645] | [0.440, 0.649] |
| # Positive, unsuppressed | 0.361 | 0.415 |
|  | [0.229, 0.494] | [0.284, 0.547] |
| # Negative | 0.500 | 0.475 |
|  | [0.433, 0.568] | [0.407, 0.543] |
| # Positive, suppressed | 0.506 | 0.524 |
|  | [0.414, 0.599] | [0.435, 0.613] |
| # Positive, unsuppressed | 0.341 | 0.397 |
|  | [0.229, 0.452] | [0.287, 0.507] |
| # Negative | 0.467 | 0.441 |
|  | [0.412, 0.521] | [0.387, 0.496] |
| # Positive, suppressed | 0.477 | 0.504 |
|  | [0.400, 0.554] | [0.429, 0.579] |
| # Positive, unsuppressed | 0.320 | 0.378 |
|  | [0.228, 0.412] | [0.288, 0.469] |
| # Negative | 0.433 | 0.408 |
|  | [0.390, 0.475] | [0.366, 0.451] |
| # Positive, suppressed | 0.448 | 0.484 |
|  | [0.384, 0.511] | [0.421, 0.548] |
| # Positive, unsuppressed | 0.300 | 0.360 |
|  | [0.224, 0.376] | [0.285, 0.434] |
| # Negative | 0.399 | 0.375 |
|  | [0.367, 0.431] | [0.343, 0.407] |
| # Positive, suppressed | 0.418 | 0.464 |
|  | [0.363, 0.473] | [0.408, 0.520] |
| # Positive, unsuppressed | 0.279 | 0.341 |
|  | [0.214, 0.344] | [0.276, 0.407] |
| # Negative | 0.365 | 0.342 |
|  | [0.339, 0.391] | [0.316, 0.367] |
| # Positive, suppressed | 0.389 | 0.444 |
|  | [0.335, 0.442] | [0.390, 0.498] |
| # Positive, unsuppressed | 0.259 | 0.323 |
|  | [0.196, 0.321] | [0.257, 0.389] |
| # Negative | 0.331 | 0.309 |
|  | [0.305, 0.357] | [0.282, 0.335] |
| # Positive, suppressed | 0.359 | 0.424 |
|  | [0.300, 0.419] | [0.365, 0.483] |
| # Positive, unsuppressed | 0.238 | 0.304 |
|  | [0.169, 0.307] | [0.229, 0.380] |
| # Negative | 0.297 | 0.275 |
|  | [0.264, 0.331] | [0.241, 0.310] |
| # Positive, suppressed | 0.330 | 0.404 |
|  | [0.260, 0.400] | [0.336, 0.472] |
| # Positive, unsuppressed | 0.218 | 0.286 |
|  | [0.135, 0.300] | [0.194, 0.377] |
| Number of observations | 9,198 | 8,511 |

*Model 1 adjusts for age and practice effects. Model 2 adjusts for all covariates. The table displays the predicted episodic memory scores at yearly intervals since baseline.*
