# Supplementary material for: Episodic memory trajectories of older adults with and without HIV: A longitudinal population-based study in rural South Africa
Source: PLOS Glob Public Health. 2026 Jun 26;6(6):e0006572. doi: 10.1371/journal.pgph.0006572 (PMC13309049; doi:10.1371/journal.pgph.0006572)
Supplement: S4 Table — (DOCX) [file pgph.0006572.s004.docx]

S4 Table: Regression coefficients of Model 2 with two additional mental health covariates

|  | **Model 2** |
| --- | --- |
| **HIV Status** | |
| Negative | *Ref.* |
| Positive, suppressed | 0.033 |
|  | [-0.049, 0.115] |
|  | *0.43* |
| Positive, unsuppressed | -0.095 |
|  | [-0.211, 0.020] |
|  | *0.11* |
| **Years since Wave 1** | |
|  | -0.033 |
|  | [-0.048, -0.019] |
|  | *0.00* |
| **HIV status x Years since Wave 1** | |
| Negative x Months | *Ref.* |
| Positive, suppressed x Months | 0.013 |
|  | [-0.002, 0.028] |
|  | *0.08* |
| Positive, unsuppressed x Months | 0.014 |
|  | [-0.009, 0.038] |
|  | *0.23* |
| **Continuous control variables** | |
| Age | -0.019 |
|  | [-0.022, -0.017] |
|  | *0.00* |
| CESD-8 Score | -0.011 |
|  | [-0.026, 0.003] |
|  | *0.11* |
| Post-traumatic stress disorder score | 0.022 |
|  | [0.007, 0.037] |
|  | *0.00* |
| Emotional well-being score | 0.023 |
|  | [0.011, 0.034] |
|  | *0.00* |
| **Practice effect** | |
| No | *Ref.* |
| Yes | -0.467 |
|  | [-0.550, -0.385] |
|  | *0.00* |
| **Sex** | |
| Male | *Ref.* |
| Female | -0.027 |
|  | [-0.075, 0.020] |
|  | *0.26* |

| **Education** | |
| --- | --- |
| No education | *Ref.* |
| Primary (1-7 yr) | 0.078 |
|  | [0.021, 0.135] |
|  | *0.01* |
| Secondary (8-12 yr) | 0.246 |
|  | [0.164, 0.329] |
|  | *0.00* |
| Secondary or more (12+ yr) | 0.403 |
|  | [0.307, 0.499] |
|  | *0.00* |
| **Wealth quintile** | |
| Quintile 1 | *Ref.* |
| Quintile 2 | -0.016 |
|  | [-0.081, 0.048] |
|  | *0.62* |
| Quintile 3 | 0.055 |
|  | [-0.013, 0.123] |
|  | *0.12* |
| Quintile 4 | 0.071 |
|  | [0.006, 0.136] |
|  | *0.03* |
| Quintile 5 | 0.116 |
|  | [0.046, 0.187] |
|  | *0.00* |
| **Marital status** | |
| Not married | *Ref.* |
| Married | 0.062 |
|  | [0.018, 0.105] |
|  | *0.01* |
| **Current smoker** | |
| No | *Ref.* |
| Yes | -0.033 |
|  | [-0.114, 0.048] |
|  | *0.43* |
| **Consumes alcohol** | |
| No | *Ref.* |
| Yes | -0.104 |
|  | [-0.160, -0.048] |
|  | *0.00* |
| **Has hypertension** | |
| No | *Ref.* |
| Yes | 0.010 |
|  | [-0.033, 0.052] |
|  | *0.66* |

| **Has diabetes** | |
| --- | --- |
| No | *Ref.* |
| Yes | -0.045 |
|  | [-0.113, 0.024] |
|  | *0.20* |
| **Literate** | |
| No | *Ref.* |
| Yes | 0.216 |
|  | [0.162, 0.270] |
|  | *0.00* |
| **Born in South Africa** | |
| No | *Ref.* |
| Yes | 0.052 |
|  | [0.001, 0.103] |
|  | *0.04* |
| **Self-rated child health** | |
| Very good, good | *Ref.* |
| Moderate, bad, very bad | 0.155 |
|  | [0.096, 0.214] |
|  | *0.00* |
| **Health service use** | |
| No | *Ref.* |
| Yes | -0.098 |
|  | [-0.140, -0.056] |
|  | *0.00* |
| **Intercept** | |
|  | 1.152 |
|  | [0.945, 1.358] |
|  | *0.00* |
| ***N*** | ***8,508*** |

*Note: The table displays the coefficients of the generalized estimation equation regression models. 95% confidence intervals are displayed in squared brackets and p-values in italics.*

*Abbreviations: CES-D: Center for Epidemiologic Studies-Depression; CI = confidence interval; Ref = reference category*
