# Supplementary material for: Episodic memory trajectories of older adults with and without HIV: A longitudinal population-based study in rural South Africa
Source: PLOS Glob Public Health. 2026 Jun 26;6(6):e0006572. doi: 10.1371/journal.pgph.0006572 (PMC13309049; doi:10.1371/journal.pgph.0006572)
Supplement: S2 Fig — (DOCX) [file pgph.0006572.s006.docx]

S2 Fig: Mixed-effects model


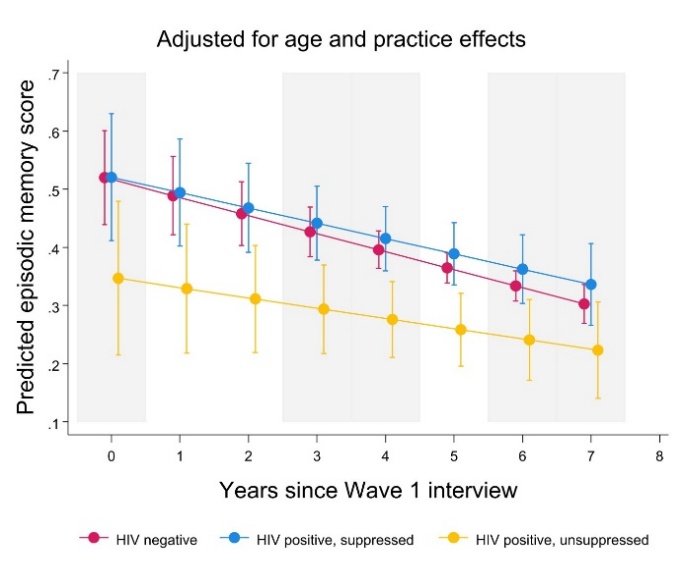

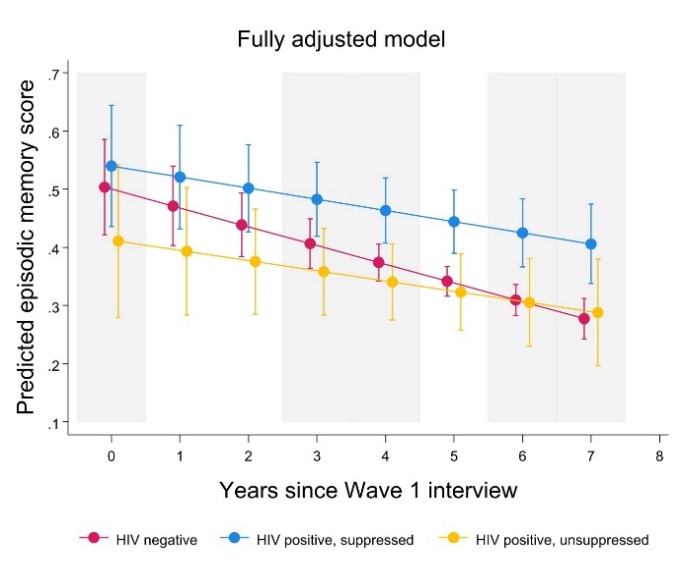


*This figure displays the predicted episodic memory score resulting from mixed-effects model estimation with 95% confidence intervals at yearly intervals for the three HIV status groups. Survey data were collected in the grey-shaded years.*
