# Supplementary material for: Episodic memory trajectories of older adults with and without HIV: A longitudinal population-based study in rural South Africa
Source: PLOS Glob Public Health. 2026 Jun 26;6(6):e0006572. doi: 10.1371/journal.pgph.0006572 (PMC13309049; doi:10.1371/journal.pgph.0006572)
Supplement: Attachment — Submitted filename: PointByPointResponse_2026-02-10.docx [file pgph.0006572.s008.docx]

***Journal Requirements:***

Comment: Please provide additional details regarding participant consent. If you are reporting a retrospective study of medical records or archived samples, please ensure that you have discussed whether all data were fully anonymized before you accessed them and/or whether the IRB or ethics committee waived the requirement for informed consent. If patients provided informed written consent to have data from their medical records used in research, please include this information.

Response: We have now included additional information on the consent procedure in the manuscript (page 6, line 143f), as follows:

*“Written informed consent was obtained from all potential participants. If a participant could not read, they signed the consent form with a fingerprint, while a witness was present.”*

Comment: Please ensure that your Ethics Statement is available in its entirety at the beginning of your Methods section, under a subheading 'Ethics Statement'.

Response: We have now moved the Ethics Statement section to the beginning of the Methods section (page 5, line 119ff), as follows:

*“Ethical approval*

*Ethical approval for HAALSI was obtained from the University of the Witwatersrand Human Research Ethics Committee (no. M141159), the Harvard T.H. Chan School of Public Health Office of Human Research Administration (no. 13-1608), and the Mpumalanga Provincial Research and Ethics Committee.”*

Comment: Please upload separate figure files in .tif or .eps format. Also, remove the figures from your manuscript file but keep the legends.

For more information about figure files please see our guidelines:

https://journals.plos.org/globalpublichealth/s/figures

<https://journals.plos.org/globalpublichealth/s/figures#loc-file-requirements>

Response: Thank you for this guidance. We have now removed the figures from our manuscript and submitted them as .tif files.

Comment: We have noticed that you have cited Table S1 in your manuscript. However, there are no corresponding files uploaded to the submission. Please upload them as separate files with the item type 'Supporting Information'.

If the reviewer comments include a recommendation to cite specific previously published works, please review and evaluate these publications to determine whether they are relevant and should be cited. There is no requirement to cite these works unless the editor has indicated otherwise.

Response: Thank you for this guidance. We will submit the supplementary material as a separate file rather than at the end of the manuscript.

***Reviewer #1:***

Comment: The population of older adults with HIV in sub-Saharan Africa is on an upward trend, which makes this work relevant.

Response: We thank Reviewer 1 for the positive assessment of the relevance of our study.

Comment: However, the paper has some limitations. The fact that HIV status and viral suppression were only determined at baseline and not considered during follow up is a major limitation. In lines 158-160, it is apparent that HIV status was determined during each wave. Was an effort made to ensure that the HIV negatives that were included remained negative during the follow-up period?

Response: Thank you for this important comment. We agree that HIV status conversion and fluctuations in viral suppression can have an influence on individual’s episodic memory in the long term.

In our sample of 2,308 individuals without HIV at baseline, only 43 (1.9%) contracted HIV during our observation period. Of the 471 individuals with HIV and suppressed viral load at baseline, 411 (87.3%) maintained viral suppression throughout all three survey waves and 60 (12.5%) did not achieve viral suppression in at least one follow-up wave.

We have now included a sensitivity analysis with the following groups: Always negative, always suppressed, (sometimes) unsuppressed, and converter. While there are slight differences in coefficient magnitudes, the overall conclusions of the main analysis remain valid.

We have added the following text to the methods and results sections:

*“We conducted several sensitivity analyses to test the robustness of our results. First, rather than defining HIV status at baseline, we considered that some individuals transitioned between statuses. Hence, we defined an alternative exposure that captures HIV status across all waves: always negative, always suppressed, at times or always unsuppressed, and converter from HIV negative to positive.”* (page 8, line 213ff)

*“Our results were robust to changes in model specifications. When considering HIV status across waves, we found slightly different coefficient estimates, but the overall conclusions of the main analysis remained valid (Figure S1).”* (page 8, line 289ff)

Comment: In the statistical section, the authors should justify why they used generalised estimating equations and not mixed effects linear regression models, which may be more appropriate for longitudinal data. The model that was used with the generalised estimating equations should be specified.

Response: We agree with Reviewer 1 that a mixed effects model is a commonly applied method for studies like ours. We estimated generalized estimating equations (GEE) because we used inverse probability weighting to account for selective attrition. As demonstrated by Tchetgen Tchetgen et al. (2012), GEE with an independence correlation structure provided unbiased estimates in these study set-ups. Specifically, the authors showed that regression estimates can be biased when using non-independent correlation structures, as is the case for mixed effects models, especially if attrition is not independent of the outcome. This may be the case in our analysis. We have now estimated a mixed effects model as a sensitivity analysis and provide the results in the appendix. While the coefficient estimates change slightly, the overall interpretation of the results remains unchanged.

We have added the following text to the methods and results sections:

*“Second, we estimated generalized estimating equations (GEE) with a Gaussian distribution family and identity link function, an independent correlation structure, and robust standard errors clustered at the individual-level.^29,30^ We estimated a GEE rather than a mixed effects model as the independent correlation structure yields unbiased estimates even if attrition is selective.”* (page 7, line 192ff)

*“We conducted several sensitivity analyses to test the robustness of our results. […]* *Second, we estimated a mixed effects model with random intercepts. Unlike GEE, mixed models cannot incorporate inverse probability weights, and therefore rely on the assumption that data are Missing at Random conditional on observed covariates.”* (page 8, line 213ff)

*“Our results were robust to changes in model specifications. […] Estimating a mixed effects model with random intercepts also did not alter the results (Figure Sx).”* (page 8, line 289ff)

Comment: Results- Regarding Table 1, the statistical test that was used to determine the group difference needs to be specified. All abbreviations that are used in the Tables should be defined.

Response: We thank Reviewer 1 for identifying this oversight. We have now added a note to the table that specifies the tests applied to determine significance of group differences and a list of abbreviations used.

Comment: The presentation of Table 2 can be improved by labelling the columns (confidence intervals, P-values, etc).

Response: We thank Reviewer 1 for this suggestion and have made the suggested changes.

***Reviewer #2:***

Comment: Given that the study area was characterised by food shortages, could poor nutrition be a confounder for steeper episodic memory decline in the HIV-negative population, given that poor nutrition significantly accelerates cognitive decline in old age?

Did the participants living with HIV ever receive nutritional supplements?

Response: We thank Reviewer 2 for this important comment and agree that nutrition and food shortages may lead to cognitive decline. Unfortunately, we don’t have information on nutritional supplements. Our data contain, however, three questions on food insecurity (not having money to buy enough food; going to sleep hungry; not eating for an entire day) with the answer options never (= 0), rarely (= 1), sometimes (= 2), often (= 3). We created a composite score ranging from 0 – 9 and included it as control variable. The overall pattern in the results did not change but the confidence intervals became wider and overlapped (see left panel of the figure below). This is not a result of adjusting for food insecurity but is caused by the smaller sample size as 10% of participants did not have information on food insecurity. The right panel of the figure below shows the result when estimating our original model on the subset of individuals with information on food insecurity. The results are almost identical. Thus, we prefer not to include food insecurity in our primary analysis. However, if the reviewer or editor feel strongly about this, we are happy to include the as a supplementary analysis.

Comment: What was the impact of depression, diabetes and hypertension on the study results? For instance, if a participant was depressed and had both diabetes and hypertension, how were their results analysed differently from another diagnosed with only one of these conditions? Please add more details to the statistical adjustment for potential confounders.

Response: We agree that the clustering of health conditions might have differential, negative effects on episodic memory and that to date evidence on this relationship is scarce. As the focus of our paper is to assess differences in trajectories across HIV status groups, we have included mental health, diabetes and hypertension as covariates rather than exposures in our regression model. Specifically, the regression model was set up as follows:

EpisodicMemory =β_0_ + β_1_ * HIVstatus +β_2_ * PracticeEffect + β_3_ * YearsSinceBaseline + β_4_ * HIVstatus*YearsSinceBaseline + β_5_ * Female + β_6_ * Education + β_7_ * Literate + β_8_ * WealthQuintile + β_9_ * MaritalStatus + β_10_ * Smoking + β_11_ * AlcoholUse + β_12_ * CESDscore + β_13_ * Hypertension + β_14_ * Diabetes + β_15_ * OriginCountry + β_16_ * ChildHealth + β_17_ * HealthServiceUse + ε

Table S2 in the Supplementary Material shows that the association between episodic memory and diabetes or hypertension was not statistically significant, whereas the association with the CES-D 8 score was significant.

Assessing how the clustering and interaction of other health conditions are associated with trajectories in episodic memory is an important research question that should by itself be the focus of a manuscript as it would require a different analytical approach and deserves an extensive contextualization and discussion of the results. Hence, we prefer to maintain the current focus of our paper and address the issue of multi-morbidity in a separate study.

Comment: With participants with HIV being on average 6 years younger than individuals without HIV, and advancing age a predictor of cognitive deficits, could this have skewed the results?

Response: We agree that the age difference between the groups is a crucial confounder when assessing trajectories of episodic memory. For this reason, we have included age in years in both regression models. Table S1 in the appendix shows that increasing age is significantly associated with declines in episodic memory, emphasizing the need to control for age. Consequently, the results of our paper can be interpreted as group differences in episodic memory trajectories minus the “effect” of age (and the other covariates).

***Reviewer #3:***

Comment: This paper addresses an important area of research -thus, it captures the contemporary issues and the common challenges in the HIV program, specifically the growing burden of comorbidities affecting people living with HIV. The knowledge gaps were clearly identified and well-articulated – particularly the scarcity of scientific evidence about the cognitive function trajectories in people living with HIV and are virally unsuppressed. The study aimed to clearly establish the association between HIV status and episodic memory changes.

Response: We thank Reviewer 3 for the positive assessment of our paper.

Comment: However, there is a need for information on the sampling method you used. For instance, the authors mentioned that it was a gender-stratified random sample but did not explicitly state the size of the population.

Response: We have now added additional information on the sampling strategy in the text :

*“The HDSS covered 31 villages with a combined population of 116,000. Of the 12,875 eligible individuals (aged 40 years and older and having lived in the HDSS for at least 12 months), 6,281 participants were randomly selected, of which 5,059 (2,714 women; 2,345 men) completed the interview.”* (page 5, line 136ff)

Comment: On the regression model – considering the use of longitudinal data in the analysis. perhaps there’s a need to use Cox proportional hazard model. This might be the appropriate model for the type of data that are being analysed.

Response: We thank Reviewer 3 for this suggestion and agree that Cox proportional hazard models provide valuable insights. However, these models require an outcome structured as time to an “event” when an individual moves from not having to having the outcome, which currently is not available in the data. Future work with the HAALSI cohort could leverage proportional hazards models for related cognitive outcomes such as date of new dementia diagnosis or date of first observation with cognitive impairment once these data have been collected.

Comment: The authors could explore more confounding variables that are directly linked to treatment regimen.

Response: We thank Reviewer 3 for this comment and agree that the specific regimen may be associated with memory trajectories and that a subgroup analysis among individuals with HIV could provide important insights. At the time of data collection, all individuals on ART were on the same regimen. Hence, no subgroup analysis is possible.

Comment: Perhaps, the mental health history could have been explored further.

Response: We thank Reviewer 3 for this comment. We have now included an exploratory analysis in the supplementary material that, in addition to the CES-D 8 score, includes a post-traumatic stress disorder score, and an emotional wellbeing score as covariates. The trajectories in episodic memory are very similar to those of the main analysis. However, the coefficient of the CES-D 8 score becomes non-significant, while those of the two new mental health indicators are statistically significant. As the main results do not change and the focus on the paper is on the relationship between episodic memory and HIV status, we have not included a detailed exploration on the role of mental health in the discussion. We think this is a highly interesting research question, which will best be explored in a separate study.

We have added the following text to the methods and results sections:

*“Third, we additionally included a continuous post-traumatic stress disorder score and an emotional well-being score as control variables.^35,36^ Details on how these two scores were generated are provided in Table S1.”* (page 8, line 219ff)

*“Estimating a mixed effects model with random intercepts also did not alter the results (Figure S2) nor did including two additional mental health indicators (Table S4, Figure S3).”* (page 8, line 291ff)
