# Supplementary material for: Episodic memory trajectories of older adults with and without HIV: A longitudinal population-based study in rural South Africa
Source: PLOS Glob Public Health. 2026 Jun 26;6(6):e0006572. doi: 10.1371/journal.pgph.0006572 (PMC13309049; doi:10.1371/journal.pgph.0006572)
Supplement: Attachment — Submitted filename: Reviewer Comments- March 2026.docx [file pgph.0006572.s009.docx]

**Reviewer Comments**

**Article: Episodic memory trajectories of older adults with and without HIV: A longitudinal population-based study in rural South Africa**

The authors have satisfactorily addressed all the comments.

I have no further comments.
