# Supplementary material for: Episodic memory trajectories of older adults with and without HIV: A longitudinal population-based study in rural South Africa
Source: PLOS Glob Public Health. 2026 Jun 26;6(6):e0006572. doi: 10.1371/journal.pgph.0006572 (PMC13309049; doi:10.1371/journal.pgph.0006572)
Supplement: Peer review history

# Author comment: Author response to Decision Letter 0

**DOI:** 10.1371/journal.pgph.0006572.r001

**Transfer Alert**

This paper was transferred from another journal. As a result, its full editorial history (including decision letters, peer reviews and author responses) may not be present.

5 Sep 2025

# Peer review: Decision Letter 0

**DOI:** 10.1371/journal.pgph.0006572.r002

**Authors:** Jason Morgan

14 Jan 2026

PGPH-D-25-02121

Episodic memory trajectories of older adults with and without HIV: A longitudinal population-based study in rural South Africa

PLOS Global Public Health

Dear Dr. Theilmann,

Thank you for submitting your manuscript to PLOS Global Public Health. After careful consideration, we feel that it has merit but does not fully meet PLOS Global Public Health’s publication criteria as it currently stands. Therefore, we invite you to submit a revised version of the manuscript that addresses the points raised during the review process.

Your manuscript has been assessed by three reviewers. While they indicate some interest in your work, they have raised several concerns that require your attention. Please consider these points carefully when preparing your revised manuscript and point-by-point response document.

Please submit your revised manuscript by Feb 28 2026 11:59PM. If you will need more time than this to complete your revisions, please reply to this message or contact the journal office at globalpubhealth@plos.org. When you’re ready to submit your revision, log on to https://www.editorialmanager.com/pgph/ and select the ‘Submissions Needing Revision’ folder to locate your manuscript file.

Please include the following items when submitting your revised manuscript:

- A letter that responds to each point raised by the editor and reviewer(s). You should upload this letter as a separate file labeled ‘Response to Reviewers’.
- A marked-up copy of your manuscript that highlights changes made to the original version. You should upload this as a separate file labeled ‘Revised Manuscript with Track Changes’.
- An unmarked version of your revised paper without tracked changes. You should upload this as a separate file labeled ‘Manuscript’.

Guidelines for resubmitting your figure files are available below the reviewer comments at the end of this letter.

We look forward to receiving your revised manuscript.

Kind regards,

Jason Morgan

Staff Editor

PLOS Global Public Health

Journal Requirements:

1. Please provide additional details regarding participant consent. If you are reporting a retrospective study of medical records or archived samples, please ensure that you have discussed whether all data were fully anonymized before you accessed them and/or whether the IRB or ethics committee waived the requirement for informed consent. If patients provided informed written consent to have data from their medical records used in research, please include this information.

2. Please ensure that your Ethics Statement is available in its entirety at the beginning of your Methods section, under a subheading ‘Ethics Statement’.

3. Please upload separate figure files in .tif or .eps format. Also, remove the figures from your manuscript file but keep the legends.

For more information about figure files please see our guidelines:

https://journals.plos.org/globalpublichealth/s/figures

https://journals.plos.org/globalpublichealth/s/figures#loc-file-requirements

4. We have noticed that you have cited Table S1 in your manuscript. However, there are no corresponding files uploaded to the submission. Please upload them as separate files with the item type ‘Supporting Information’.

If the reviewer comments include a recommendation to cite specific previously published works, please review and evaluate these publications to determine whether they are relevant and should be cited. There is no requirement to cite these works unless the editor has indicated otherwise.

Additional Editor Comments (if provided):

[Note: HTML markup is below. Please do not edit.]

Reviewers’ comments:

Reviewer’s Responses to Questions

**Comments to the Author**

1. Does this manuscript meet PLOS Global Public Health’s publication criteria? Is the manuscript technically sound, and do the data support the conclusions? The manuscript must describe methodologically and ethically rigorous research with conclusions that are appropriately drawn based on the data presented.

Reviewer #1: Yes

Reviewer #2: Yes

Reviewer #3: Yes

**********

2. Has the statistical analysis been performed appropriately and rigorously?

Reviewer #1: No

Reviewer #2: Yes

Reviewer #3: Yes

**********

3. Have the authors made all data underlying the findings in their manuscript fully available (please refer to the Data Availability Statement at the start of the manuscript PDF file)?

The PLOS Data policy requires authors to make all data underlying the findings described in their manuscript fully available without restriction, with rare exception. The data should be provided as part of the manuscript or its supporting information, or deposited to a public repository. For example, in addition to summary statistics, the data points behind means, medians and variance measures should be available. If there are restrictions on publicly sharing data—e.g. participant privacy or use of data from a third party—those must be specified.

Reviewer #1: Yes

Reviewer #2: Yes

Reviewer #3: Yes

**********

4. Is the manuscript presented in an intelligible fashion and written in standard English?

PLOS Global Public Health does not copyedit accepted manuscripts, so the language in submitted articles must be clear, correct, and unambiguous. Any typographical or grammatical errors should be corrected at revision, so please note any specific errors here.

Reviewer #1: Yes

Reviewer #2: Yes

Reviewer #3: Yes

**********

5. Review Comments to the Author

Please use the space provided to explain your answers to the questions above. You may also include additional comments for the author, including concerns about dual publication, research ethics, or publication ethics. (Please upload your review as an attachment if it exceeds 20,000 characters)

Reviewer #1: Reviewer Comments

Article: Episodic memory trajectories of older adults with and without HIV: A longitudinal population-based study in rural South Africa

The population of older adults with HIV in sub-Saharan Africa is on an upward trend, which makes this work relevant.

However, the paper has some limitations. The fact that HIV status and viral suppression were only determined at baseline and not considered during follow up is a major limitation. In lines 158-160, it is apparent that HIV status was determined during each wave. Was an effort made to ensure that the HIV negatives that were included remained negative during the follow-up period?

In the statistical section, the authors should justify why they used generalised estimating equations and not mixed effects linear regression models, which may be more appropriate for longitudinal data.

The model that was used with the generalised estimating equations should be specified.

Results- Regarding Table 1, the statistical test that was used to determine the group difference needs to be specified. All abbreviations that are used in the Tables should be defined.

The presentation of Table 2 can be improved by labelling the columns (confidence intervals, P-values, etc).

Reviewer #2: Given that the study area was characterised by food shortages, could poor nutrition be a confounder for steeper episodic memory decline in the HIV-negative population, given that poor nutrition significantly accelerates cognitive decline in old age?

Did the participants living with HIV ever receive nutritional supplements?

What was the impact of depression, diabetes and hypertension on the study results? For instance, if a participant was depressed and had both diabetes and hypertension, how were their results analysed differently from another diagnosed with only one of these conditions? Please add more details to the statistical adjustment for potential confounders.

With participants with HIV being on average 6 years younger than individuals without HIV, and advancing age a predictor of cognitive deficits, could this have skewed the results?

Reviewer #3: This paper addresses an important area of research -thus, it captures the contemporary issues and the common challenges in the HIV program, specifically the growing burden of comorbidities affecting people living with HIV. The knowledge gaps were clearly identified and well-articulated – particularly the scarcity of scientific evidence about the cognitive function trajectories in people living with HIV and are virally unsuppressed. The study aimed to clearly establish the association between HIV status and episodic memory changes. However, there is a need for information on the sampling method you used. For instance, the authors mentioned that it was a gender-stratified random sample but did not explicitly state the size of the population. On the regression model – considering the use of longitudinal data in the analysis. perhaps there’s a need to use Cox proportional hazard model. This might be the appropriate model for the type of data that are being analysed. The authors could explore more confounding variables that are directly linked to treatment regimen. Perhaps, the mental health history could have been explored further.

**********

6. PLOS authors have the option to publish the peer review history of their article (what does this mean?). If published, this will include your full peer review and any attached files.

**Do you want your identity to be public for this peer review?**  If you choose “no”, your identity will remain anonymous but your review may still be made public.

For information about this choice, including consent withdrawal, please see our Privacy Policy.

Reviewer #1: No

Reviewer #2: No

Reviewer #3: No

**********

[NOTE: If reviewer comments were submitted as an attachment file, they will be attached to this email and accessible via the submission site. Please log into your account, locate the manuscript record, and check for the action link “View Attachments”. If this link does not appear, there are no attachment files.]

-->Figure Resubmissions:

While revising your submission, we strongly recommend that you use PLOS’s NAAS tool (https://ngplosjournals.pagemajik.ai/artanalysis) to test your figure files. NAAS can convert your figure files to the TIFF file type and meet basic requirements (such as print size, resolution), or provide you with a report on issues that do not meet our requirements and that NAAS cannot fix.

After uploading your figures to PLOS’s NAAS tool - https://ngplosjournals.pagemajik.ai/artanalysis, NAAS will process the files provided and display the results in the “Uploaded Files” section of the page as the processing is complete. If the uploaded figures meet our requirements (or NAAS is able to fix the files to meet our requirements), the figure will be marked as “fixed” above. If NAAS is unable to fix the files, a red “failed” label will appear above. When NAAS has confirmed that the figure files meet our requirements, please download the file via the download option, and include these NAAS processed figure files when submitting your revised manuscript.

# Author comment: Author response to Decision Letter 1

**DOI:** 10.1371/journal.pgph.0006572.r003

20 Feb 2026

Attachment Submitted filename: PointByPointResponse_2026-02-10.docx

# Peer review: Decision Letter 1

**DOI:** 10.1371/journal.pgph.0006572.r004

**Authors:** Jason Morgan, Julia Robinson

14 May 2026

Episodic memory trajectories of older adults with and without HIV: A longitudinal population-based study in rural South Africa

PGPH-D-25-02121R1

Dear Mrs. Theilmann,

We are pleased to inform you that your manuscript ‘Episodic memory trajectories of older adults with and without HIV: A longitudinal population-based study in rural South Africa’ has been provisionally accepted for publication in PLOS Global Public Health.

Before your manuscript can be formally accepted you will need to complete some formatting changes, which you will receive in a follow up email. A member of our team will be in touch with a set of requests.

Please note that your manuscript will not be scheduled for publication until you have made the required changes, so a swift response is appreciated.

IMPORTANT: The editorial review process is now complete. PLOS will only permit corrections to spelling, formatting or significant scientific errors from this point onwards. Requests for major changes, or any which affect the scientific understanding of your work, will cause delays to the publication date of your manuscript.

If your institution or institutions have a press office, please notify them about your upcoming paper to help maximize its impact. If they’ll be preparing press materials, please inform our press team as soon as possible -- no later than 48 hours after receiving the formal acceptance. Your manuscript will remain under strict press embargo until 2 pm Eastern Time on the date of publication. For more information, please contact globalpubhealth@plos.org.

Thank you again for supporting Open Access publishing; we are looking forward to publishing your work in PLOS Global Public Health.

Best regards,

Julia Robinson

Executive Editor

PLOS Global Public Health

***********************************************************

Reviewer Comments (if any, and for reference):

Reviewer’s Responses to Questions

**Comments to the Author**

1. If the authors have adequately addressed your comments raised in a previous round of review and you feel that this manuscript is now acceptable for publication, you may indicate that here to bypass the “Comments to the Author” section, enter your conflict of interest statement in the “Confidential to Editor” section, and submit your “Accept” recommendation.

Reviewer #1: All comments have been addressed

**********

2. Does this manuscript meet PLOS Global Public Health’s publication criteria? Is the manuscript technically sound, and do the data support the conclusions? The manuscript must describe methodologically and ethically rigorous research with conclusions that are appropriately drawn based on the data presented.

Reviewer #1: Yes

**********

3. Has the statistical analysis been performed appropriately and rigorously?

Reviewer #1: Yes

**********

4. Have the authors made all data underlying the findings in their manuscript fully available (please refer to the Data Availability Statement at the start of the manuscript PDF file)?

The PLOS Data policy requires authors to make all data underlying the findings described in their manuscript fully available without restriction, with rare exception. The data should be provided as part of the manuscript or its supporting information, or deposited to a public repository. For example, in addition to summary statistics, the data points behind means, medians and variance measures should be available. If there are restrictions on publicly sharing data—e.g. participant privacy or use of data from a third party—those must be specified.

Reviewer #1: Yes

**********

5. Is the manuscript presented in an intelligible fashion and written in standard English?

PLOS Global Public Health does not copyedit accepted manuscripts, so the language in submitted articles must be clear, correct, and unambiguous. Any typographical or grammatical errors should be corrected at revision, so please note any specific errors here.

Reviewer #1: Yes

**********

6. Review Comments to the Author

Please use the space provided to explain your answers to the questions above. You may also include additional comments for the author, including concerns about dual publication, research ethics, or publication ethics. (Please upload your review as an attachment if it exceeds 20,000 characters)

Reviewer #1: The authors have satisfactorily addressed all the comments.

I have no further comments.

**********

7. PLOS authors have the option to publish the peer review history of their article (what does this mean?). If published, this will include your full peer review and any attached files.

**Do you want your identity to be public for this peer review?**  If you choose “no”, your identity will remain anonymous but your review may still be made public.

For information about this choice, including consent withdrawal, please see our Privacy Policy.

Reviewer #1: No

**********

Attachment Submitted filename: Reviewer Comments- March 2026.docx
